# Complete genome sequence of *Photobacterium piscicola* strain WVL24019 isolated from a freshwater hatchery-raised rainbow trout (*Oncorhynchus mykiss*)

**DOI:** 10.1128/mra.01360-24

**Published:** 2025-04-21

**Authors:** Pacharapong Khrongsee, Apryle Panyi, Kuttichantran Subramaniam, Joseph M. Groff

**Affiliations:** 1 Department of Infectious Diseases and Immunology, College of Veterinary Medicine, University of Florida 3463, Gainesville, Florida, USA; 2 Emerging Pathogens Institute, University of Florida 3463, Gainesville, Florida, USA; 3 Faculty of Veterinary Science, Prince of Songkla University, Hatyai, Songkhla, Thailand; 4 Animal Health Diagnostic Laboratory, New Jersey Department of Agriculture, Ewing, New Jersey, USA; 5 Office of Fish and Wildlife Health & Forensics, NJ Fish & Wildlife, New Jersey Department of Environmental Protection, Oxford, New Jersey, USA; University of Southern California, Los Angeles, California, USA

**Keywords:** photobacteria, *Photobacterium piscicola*, complete genome sequence, freshwater, rainbow trout, *Oncorhynchus mykiss*

## Abstract

This report describes the complete genome sequence of the non-bioluminescent marine bacterium *Photobacterium piscicola* strain WVL24019, isolated from a one-year freshwater hatchery-raised rainbow trout in a non-recirculating system supplied by aquiferous water. The genome was sequenced using Oxford Nanopore and Illumina NovaSeq technologies, resulting in two complete circular chromosomes.

## ANNOUNCEMENT

Members of the genus *Photobacterium* (family *Vibrionaceae*) are primarily marine bacteria with roles as luminescent symbionts, pathogens, or saprotrophs (1–4). *Photobacterium piscicola* was first isolated from marine fish, but its complete genome has not been documented (5). Herein is the complete genome sequence of *P. piscicola* strain WVL24019, which was isolated from a freshwater fish expanding the known habitat range of this species.

Strain WVL24019 was isolated from the kidney of an apparently healthy, 1-year freshwater hatchery-raised rainbow trout (*Oncorhynchus mykiss*) housed in a non-recirculating aquifer-fed system maintained at 12℃–13°C in northwestern New Jersey, USA. The kidney was cultured with an inoculating loop prior to streaking on TSA and incubation at 15°C. The isolate was initially identified as *Vibrio anguillarum* using the Sensititre Aris 2X ID/AST Diagnostic System (Thermo Fisher). The isolate was subcultured on TSA at 15°C for DNA extraction and subsequent genetic sequencing.

The sample was obtained during routine health monitoring following the fish health policy for state hatcheries: https://dep.nj.gov/njfw/fishing/freshwater/health-of-hatchery-fish/.

Genomic DNA was extracted using the NucleoSpin Microbial DNA MiniKit (Macherey-Nagel) according to the manufacturer’s instructions and was subsequently used to prepare both Oxford Nanopore Technology (ONT) and Illumina libraries. ONT sequencing was conducted on a GridION sequencer with R10.4.1 flow cell using the PCR-free ONT Ligation Sequencing Kit (SQK-NBD114.24) and NEBNext® Companion Module (E7180L) protocols. No additional DNA fragmentation or size selection was performed. Basecalling, demultiplexing, and adapter trimming were performed with Guppy v6.5.7 in superaccurate mode (SUP). ONT sequencing generated 6,996,684 reads with an average read length of 1,549 bp. After filtering for a minimum length of 2,000 bp using CLC Genomics Workbench v20.0.4, the final count of reads is 1,324,265, with an average length of 4,524 bp. Genome assembly was performed using Canu v2.2, generating two circular chromosome scaffolds at an average coverage of 198.75× (6). All software was used with default parameters unless otherwise specified.

Short-read sequencing was performed on the Illumina NovaSeqX Plus platform using libraries prepared with the Illumina DNA Prep Kit and IDT 10 bp unique dual indices, targeting an insert size of 280 bp. Paired-end sequencing (2 × 151 bp) generated 3,081,796 reads. Quality control and adapter trimming were performed with bcl-convert v4.2.4, and the reads were aligned with the genome scaffolds using Bowtie2 v2.4.2 (7). Pilon v1.23 was used to correct the draft assembly, achieving an average coverage of 99.99× (8). Genome annotation was performed using the NCBI prokaryotic genome annotation pipeline v6.9 (9). The final genome assembly was manually scrutinized, and the overlapping ends were trimmed in CLC Genomics Workbench v20.0.4 to confirm completeness.

**Fig 1:**
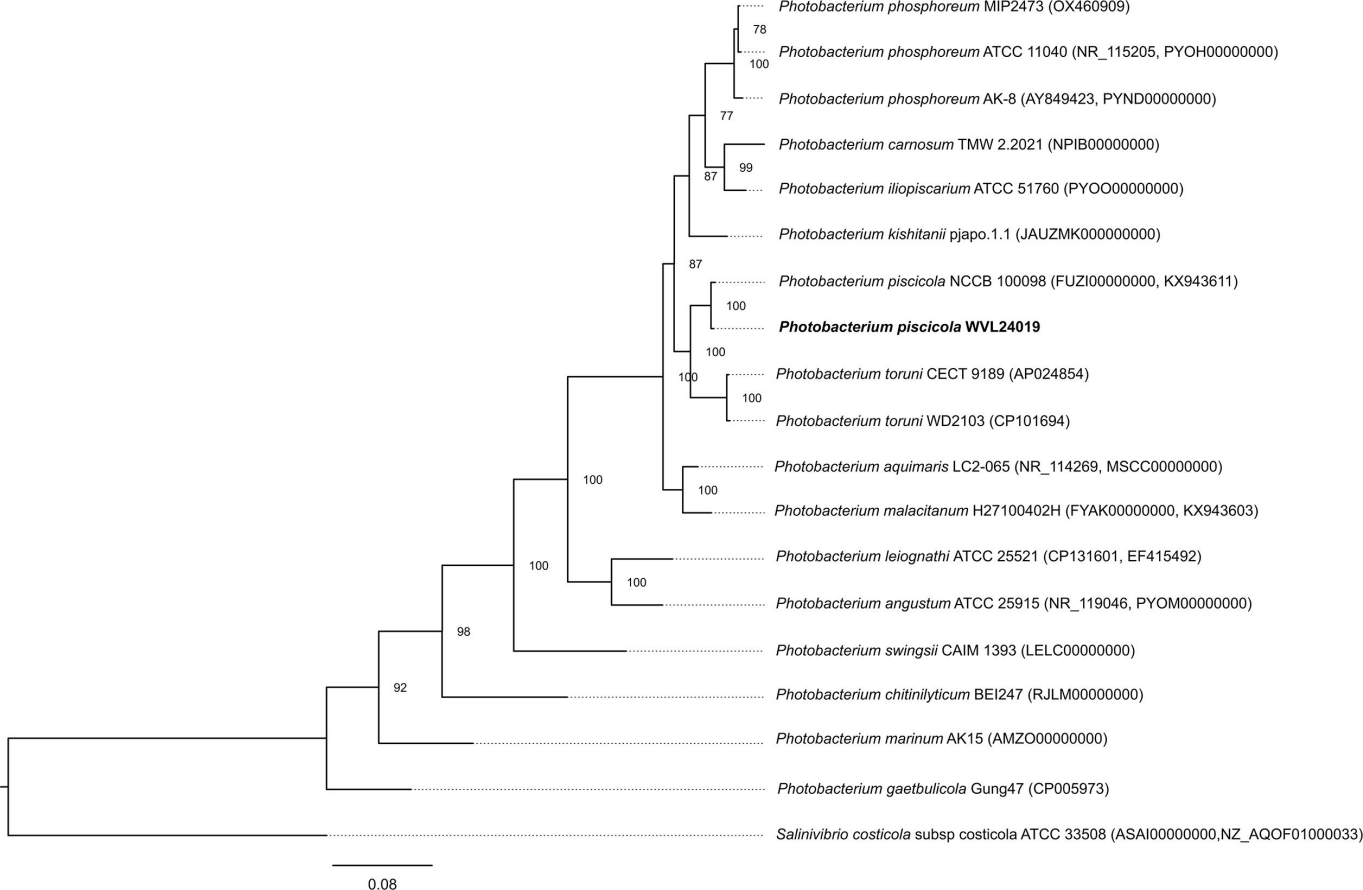
Maximum likelihood phylogram depicting the relationship of the *Photobacterium piscicola* strain WVL24019 to 17 members in the genus *Photobacterium* and *Salinivibrio costicola* subsp. costicola (outgroup) based on concatenated sequences of the *16S rRNA*, *gyrB*, *gapA*, *topA*, *ftsZ*, and *mreB* housekeeping genes. Bootstrap values (*n* = 1000) are shown at the branch nodes to indicate support for each clade.

The assembled genome consists of two circular chromosomes: 3,141,886 bp (G+C content, 40.41%) and 1,332,937 bp (G+C content, 37.64%); 3,800 coding sequences, 232 tRNA genes, and 77 rRNA genes were annotated (Table 1). No *lux* genes responsible for bioluminescence were detected. Species identification was confirmed using the Type Strain Genome Server (10) and phylogenetic analysis based on concatenated housekeeping genes (16S rRNA, *gyrB*, *gapA*, *topA*, *ftsZ*, *mreB*; Fig. 1) as previously described for *Photobacterium* spp. (11, 12). Both analyses confirmed strain WVL24019 as *P. piscicola*.

**TABLE 1:** Genome annotation features of *Photobacterium piscicola* strain WVL24019

Genome feature	Value
ONT sequencing	
No. of reads	6,996,684
No. of reads after 2,000 bp filter	1,324,265
Avg. coverage	198.75×
Illumina sequencing	
No. of reads	3,081,796
No. of base	454,949,884
No. of base change after Pilon-corrected draft assemblies	229 nt
Avg. coverage	99.99×
Assembly	
Genome structure	Two chromosomes
Chromosome 1 size (bp)	3,141,886
Chromosome 2 size (bp)	1,332,937
GC content (Chromosome 1)	40.41%
GC content (Chromosome 2)	37.64%
No. of coding sequences (CDSs)	3,800
No. of tRNA	232
No. of rRNA	77

## Data Availability

The complete genome sequence of *Photobacterium piscicola* strain WVL24019 was deposited at NCBI GenBank under BioProject accession number PRJNA1191407, Biosample accession number SAMN45078994, and GenBank accession numbers CP175534 (Chromosome I) and CP175535 (Chromosome II). The raw reads were deposited in the Sequence Read Archive under the accession numbers SRR31649280 (Oxford Nanopore) and SRR31649281 (Illumina).
